# Linking methanotroph phenotypes to genotypes using a simple spatially resolved model ecosystem

**DOI:** 10.1093/ismejo/wrae060

**Published:** 2024-04-16

**Authors:** Delaney G Beals, Aaron W Puri

**Affiliations:** Department of Chemistry and the Henry Eyring Center for Cell and Genome Science, University of Utah, Salt Lake City, UT 84112, United States; Department of Chemistry and the Henry Eyring Center for Cell and Genome Science, University of Utah, Salt Lake City, UT 84112, United States

**Keywords:** methanotroph, methane, model ecosystem, phenotypic heterogeneity

## Abstract

Connecting genes to phenotypic traits in bacteria is often challenging because of a lack of environmental context in laboratory settings. Laboratory-based model ecosystems offer a means to better account for environmental conditions compared with standard planktonic cultures and can help link genotypes and phenotypes. Here, we present a simple, cost-effective, laboratory-based model ecosystem to study aerobic methane-oxidizing bacteria (methanotrophs) within the methane-oxygen counter gradient typically found in the natural environment of these organisms. Culturing the methanotroph *Methylomonas* sp. strain LW13 in this system resulted in the formation of a distinct horizontal band at the intersection of the counter gradient, which we discovered was not due to increased numbers of bacteria at this location but instead to an increased amount of polysaccharides. We also discovered that different methanotrophic taxa form polysaccharide bands with distinct locations and morphologies when grown in the methane-oxygen counter gradient. By comparing transcriptomic data from LW13 growing within and surrounding this band, we identified genes upregulated within the band and validated their involvement in growth and band formation within the model ecosystem using knockout strains. Notably, deletion of these genes did not negatively affect growth using standard planktonic culturing methods. This work highlights the use of a laboratory-based model ecosystem that more closely mimics the natural environment to uncover bacterial phenotypes missing from standard laboratory conditions, and to link these phenotypes with their genetic determinants.

## Introduction

Despite the explosion in availability of bacterial genomic data, linking genes with organismal phenotypes remains difficult. One reason is that assigning gene functions can be hindered by a lack of ecological context in the lab, as bacteria are removed from the environments where they evolved. To address this, researchers have developed various strategies to reintegrate environmental factors and spatial structure into laboratory cultures [1–3]. Among these strategies, laboratory-based model ecosystems act as a powerful tool that can be used to more closely recapitulate the natural environment than simple planktonic cultures while still remaining experimentally tractable [4, 5]. Model ecosystems are especially valuable for studying microbes found at oxic-anoxic interfaces, like those isolated from soils and sediments. In these settings, the distribution and behavior of microbes are closely aligned with physiochemical changes, particularly the transition from oxygenated to non-oxygenated environments [6]. One such example is aerobic methane-oxidizing bacteria (methanotrophs), which play important roles in biogeochemical cycling, bioremediation, and sequestration of the potent greenhouse gas methane [7–9]. These bacteria require methane as a carbon and energy source, and molecular oxygen for respiration and as an electron acceptor and atom donor in the methane monooxygenase reaction [10].

In the environment, oxygen diffuses into soils and sediments from the atmosphere or water column above, and methane diffuses up from anaerobic decomposition processes below, resulting in opposing gradients of oxygen and methane ideal for methanotrophs [6, 11–13]. However, the oxygen-methane counter gradient is dynamic, as methane concentrations can vary depending on the availability of labile carbon in the soil, and oxygen can be depleted by increased activity by oxygen-respiring organisms [14]. To facilitate methane oxidation in micro-oxic conditions, methanotrophs employ a variety of metabolic strategies depending on their phylogeny. Many aerobic methanotrophs possess hemerythrin, a non-heme oxygen-binding transporter protein that scavenges and transports oxygen to the particulate methane monooxygenase enzyme used in the first step of methane oxidation [15]. Additionally, certain methanotrophs possess high affinity cytochromes to facilitate methane oxidation at low oxygen concentrations (e.g. *Methylobacter* spp.) [13], and some can couple this process with nitrate reduction as an energy conservation strategy (e.g. *Methylomonas denitrificans*) [16]. Consistent with these diverse metabolic strategies, studies using methane enrichments of sediment have repeatedly observed dynamic population flux amongst methanotrophic communities in response to shifting oxygen and methane availability [17, 18].

Replicating aspects of the chemical and physical environment in which methanotrophs exist is necessary for effectively studying their physiology and behavior. However, in the laboratory, these organisms have primarily been studied using homogenous planktonic cultures that lack spatial heterogeneity. This practice is likely impeding our ability to not only understand the molecular details of methanotroph physiology but also to optimize these details for industrial processes and climate change mitigation [19, 20]. Additionally, culturing methanotrophs within a spatially resolved model ecosystem may help uncover phenotypes that are otherwise undetectable in planktonic cultures. Previously, researchers demonstrated that growing methanotrophs within opposing gradients of methane and oxygen in the lab is feasible using a variety of methods. In some examples, soil was suspended on a PTFE membrane that allowed gas exchange with the methane supply below [11, 21, 22]. In other cases, sediment was added to semi-solid agarose and exposed to opposing chambers of air and methane [23, 24]. To our knowledge, these systems have been primarily used to isolate and classify methanotrophs from mixed methane-oxidizing consortia and have not yet been used to collect molecular-level information about the physiology of methanotrophs under opposing methane and oxygen gradients.

Inspired by a previously published methane-taxis assay [25], we created a simple, inexpensive, laboratory-based model ecosystem using a disposable syringe that enables researchers to culture methanotrophs in a spatially resolved context. The system, called the gradient syringe, replicates the steep methane and oxygen counter gradients that are characteristic of the native environment of methanotrophs. We used this system to study pure cultures of methanotrophic soil isolates and examined their distribution, phenotypes, and cellular activity across the gas counter gradient. The results showed heterogenous physiological responses of methanotrophs and revealed new connections between a gradient syringe-specific phenotype and the genes involved.

## Materials and Methods

### Strain growth

Strains used in this study were previously isolated from Lake Washington, Seattle, USA [26] and are listed in Table S1. Liquid cultures of methanotrophic strains were grown in an atmosphere of 50% (v/v) methane in air in liquid nitrate mineral salts (NMS) medium containing 0.2 g/l MgSO_4_·7H_2_O, 0.2 g/l CaCl_2_·6H_2_O, 1 g/l KNO_3_, and 30 μM LaCl_3_ as well as 1× trace elements; 500× trace elements contain 1.0 g/l Na_2_-EDTA, 2.0 g/l FeSO_4_·7H_2_O, 0.8 g/l ZnSO_4_·7H_2_O, 0.03 g/l MnCl_2_·4H_2_O, 0.03 g/l H_3_BO3, 0.2 g/l CoCl_2_·6H_2_O, 0.6 g/l CuCl_2_·2H_2_O, 0.02 g/l NiCl_2_·6H_2_O, and 0.05 g/l Na_2_MoO·2H_2_O. A final concentration of 5.8-mM phosphate buffer (pH 6.8) was added before use. Liquid cultures were grown in 18 × 150 mm glass tubes sealed with rubber stoppers and aluminum seals, and were shaken at 200 rpm at room temperature.

### Preparation of gradient syringes

Liquid cultures of methanotrophs in exponential-phase growth (OD_600_ ~ 0.5) were used to inoculate gradient syringes. Cells were pelleted and resuspended in sterile water to reach an approximate OD_600_ of 1.0. For each 10-ml syringe, 1 ml of concentrated cells was mixed with 5 ml NMS and 4 ml molten agarose (0.5% m/v, cooled to 55°C). The mixture was slowly poured to the 8 ml marking of a 10-ml plastic Luer-Lok tip syringe (BD) fitted with a sterile PTFE filter tip (Titan3 PTFE [Hydrophobic] Syringe Filter 0.2 μm, 4 mm, Thermo Scientific). Solidified syringes were capped with a sterile 20-mm rubber butyl stopper (Chemglass Life Sciences) and wrapped with lab tape for labeling. Inlet and outlet needles (23 gauge, BD) were pierced through the rubber stopper, and 20 ml of 100% CH_4_ was flushed through the syringe headspace. Syringes were flushed daily with methane and incubated with the PTFE filter pointing up at 18°C.

### Gas analysis of gradient syringes

The dissolved oxygen within the gradient syringe was measured using a Clark-type microelectrode (Ox50, Unisense) at 0.1-mm intervals. Immediately before measurement, the tip of a syringe was sliced off with a razor and the syringe fastened to a syringe pump (NE-1000, New Era Pump Systems Inc.) that moved the agarose plug toward the microelectrode at a rate of 1 ml/min (0.6 cm/min). Measurements were recorded and converted to μmol/l using the Unisense Logger software. The relative methane concentration in LW13-containing and cell-free gradient syringe agarose were analyzed using a gas chromatograph-flame ionization detector. Immediately before extrusion, the syringe filter was replaced with a two-way stopcock fitted with a 23-gauge needle and the syringe rubber stopper was quickly exchanged with a syringe plunger. Eight 1-ml agarose aliquots were then added to different evacuated 12-ml Exetainer vials (Labco) and allowed to equilibrate at room temperature for 1 h. Prior to injecting 500 μl of vial headspace into a 6890N gas chromatograph (Agilent), sample vials were cracked open and immediately resealed to equilibrate the samples to atmospheric pressure. A calibration curve derived from CH_4_ standards (Supelco) was used to convert peak area (pA^*^min) to μmol/l.

### Counting of methanotroph cells in gradient syringes

For flow cytometry of LW13-containing syringes, after 7 days of growth, each 1-ml aliquot of extruded agarose was diluted and homogenized with the addition of 0.75 ml of 0.85% (m/v) NaCl in water. Aliquots were further diluted 1:10 with the salt solution before staining with 3 μl of a 1:1 mixture of SYTO9 and propidium iodide (LIVE/DEAD BacLight Bacterial Viability Kit, Invitrogen) and incubated in the dark at room temperature for 15 min. Samples were analyzed with a CytoFLEX flow cytometer (Beckman Coulter) with parameters: fluidics: slow; excitation: 470 nm; emission: 490–700 nm. Stained aliquots from cell-free syringes and 70% (v/v) ethanol-treated aliquots were used as negative controls to determine voltage gates. To determine the cells per ml of agarose, 10 μl of microsphere counting beads (Invitrogen) were added to each sample prior to flow cytometry analysis and used to normalize the volume of sample to the number of cell events per agarose segment. Relative cell diameter was determined by comparing the median forward scatter signal of gated cells between agarose segments.

To determine the concentration of viable cells after cultivation in the gradient syringe, inoculated agarose was extruded in 1-ml aliquots through a 23-gauge sterile needle using the accompanying syringe plunger. Agarose was diluted with 1-ml sterile water and vortexed to homogenize the samples and aid in pipetting. Samples were serially diluted 10-fold in sterile water before spotting onto solid NMS agar plates. Plates were incubated in sealed jars under 40% methane in air and grown at 18°C. Bacterial colonies were counted and used to calculate the colony forming units per milliliter (CFUs/ml).

### Polysaccharide detection assay

Agarose segments (1 ml) were extruded into 2-ml microcentrifuge tubes and mixed with 1 ml of a 1% (m/v) Na_2_CO_3_ solution in water. Samples were heated to 80°C for 30 min with vortexing every 5–10 min, followed by centrifugation at 4000× g at 4°C for 20 min. The supernatant was collected and combined with three volumes of 100% ethanol and incubated at −20°C overnight. The ethanol-precipitated polysaccharides were collected by centrifugation at 16 100× g at 4°C for 30 min, and the air-dried pellet was resuspended in 100 μl of Milli-Q water. The relative polysaccharide content of each agarose segment was measured using a phenol-sulfuric acid colorimetric assay [27–29]. Briefly, 50 μl of resuspended extract was combined with 150-μl concentrated sulfuric acid and 30 μl of 5% (v/v) phenol in water in a clear 96-well plate. The absorbance at 490 nm was measured using a microplate reader, and the relative polysaccharide content of individual agarose segments was calculated as a percentage of the absorbance at the agarose segment closest to the PTFE filter.

### Protein and DNA concentration assays

To determine the concentration of total protein in LW13-inoculated syringes, eight 1-ml aliquots were extruded through a 23-gauge needle into individual microcentrifuge tubes and 100 μl of each were transferred to test tubes. We measured the total protein concentration following the test tube procedure of the Pierce BCA Protein Assay Kit (ThermoScientific). Albumin BSA standards were prepared using agarose extruded from a sterile syringe as the diluent. To measure the extracellular DNA concentration of LW13-inoculated syringes, 20 μl of extruded agarose aliquots were transferred to 0.2-ml microcentrifuge tubes. DNA concentrations were measured using the Qubit 1× dsDNA High Sensitivity Assay Kit (Invitrogen) following the manufacturer’s protocol and read on a Qubit 4 Fluorometer (Invitrogen).

### RNA preparation, RNA-seq, and data analysis

For each of three independent replicates, eight *Methylomonas* sp. LW13-inoculated syringes were incubated for 3 days to reach exponential growth. Agarose segments (2 ml) positioned above, at, and below the polysaccharide band were extruded from each syringe through a 23-gauge needle into microcentrifuge tubes. Samples were frozen at −80°C until RNA extraction. Thawed aliquots were centrifuged at 21 000× g at 4°C for 15 min. After removal of the supernatant, 600 μl of pre-warmed (65°C) CTAB extraction buffer was added. The CTAB buffer was composed of 2% (m/v) CTAB, 2% (m/v) polyvinylpyrrolidone (PVP 40), 1.4 M NaCl, 100 mM Tris–HCl (pH 8.0), 20 mM EDTA, and 1% (v/v) beta-mercaptoethanol in RNase-free water [30]. Zirconia glass beads were added, and samples were homogenized for 3 min at 30 Hz/s using a bead beater. Samples were extracted twice with 600-μl chloroform:isoamyl alcohol (24:1), and the aqueous phase was mixed with isopropanol and incubated overnight at −20°C. Precipitates were centrifuged at 16 100× g at 4°C for 30 min and pellets were washed twice with cold 75% (v/v) ethanol made with DEPC water. Air dried pellets were dissolved in 100-μl DEPC water and treated with DNase I (Ambion) at 37°C for 30 min. The DNase I was inactivated by extraction with three volumes of acid phenol:chloroform:IAA (125:24:1, pH 4.5) before pooling RNA from the same segment across all eight syringes in an overnight precipitation at −20°C in 1 volume isopropanol and 0.8 M LiCl. Precipitates were washed twice with cold 70% (v/v) ethanol made with DEPC water, air dried, and resuspended in 50-μl DEPC water. Samples were re-purified using RNA Clean & Concentrator-5 (Zymo Research) to remove small RNAs. To confirm RNA samples did not contain residual DNA, purified RNA was used as the template for PCR amplification using universal bacterial 16S rRNA gene primers 27F/1492R. The PCR product was checked using agarose gel electrophoresis against positive (genomic DNA) and negative (nuclease-free water) controls.

Library preparation and sequencing were performed by the High-Throughput Genomics Shared Resource Core at the University of Utah. cDNA libraries were prepared using a NEBNext Ultra II Directional RNA Library Prep Kit with rRNA Depletion (New England Biolabs) and sequenced using a NovaSeq 6000 System (Illumina) with 150 × 150 cycle paired end sequence run. RNA sequencing data analysis was conducted using a KBASE workflow [31]. Reads quality was assessed using FastQC v0.11.9, trimmed with Trimmomatic v0.36 [32], aligned using HISAT2 v2.1.0 [33], transcripts assembled with StringTie v2.1.5 [34], and differentially expressed genes identified using DESeq2 v1.20.0 [35].

### Genetic manipulation in *Methylomonas* sp. LW13

Plasmids and constructs used in this study are listed in Tables S1 and S2, and primers are listed in Table S3. All genetic manipulation of LW13 was carried out at 30°C under 40% (v/v) methane in air.

#### Deletion mutant construction

Genetic manipulations were accomplished by electroporating PCR amplification-generated constructs into LW13, followed by the selection of chromosomal recombinants, following established methods [36]. Briefly, the kanamycin resistance gene cassette (amplified from plasmid pCM433kanT [37]) and flanking regions of the target gene were assembled using overlap extension PCR. The assembled fragment was then amplified using nested primers, separated on a 1% (m/v) TAE agarose gel, and purified using a GeneJet Gel Purification Kit (ThermoFisher). After confirming correct assembly via Sanger sequencing, the fragment was electroporated directly into LW13 cells. Post-electroporation, cells were cultured overnight in NMS medium without antibiotics, and then transferred to kanamycin-containing plates (50 μg/ml). Single colonies were selected, and disruption of the gene of interest with the kanamycin resistance cassette was confirmed through colony PCR using OneTaq 2X Master Mix with Standard Buffer (New England Biolabs) with primers specific to the assembled construct.

#### Complementation of genes into mutant strains

Using overlap extension PCR, ~3000 bp sequences containing the gene of interest were constructed and electroporated into LW13 mutants. These constructs included the gene of interest under the bacterial promoter *nptII* [38], along with a zeocin resistance gene cassette directly downstream of the gene to be complemented. Flanking either end of this construct were homologous flanks targeting a distal insertion site in the LW13 genome that was previously used for complementation in this strain [39]. Full-length DNA segments were excised after separation on a 1% TAE agarose gel and purified using a GeneJet Gel Purification Kit (ThermoFisher). After introduction of the segments via electroporation, successful recombinants were selected on NMS media containing 20-μg/ml zeocin and confirmed by colony PCR.

## Results

### Different methanotrophic taxa form horizontal bands with distinct locations and morphologies when grown in the gradient syringe model ecosystem

**Figure 1:**
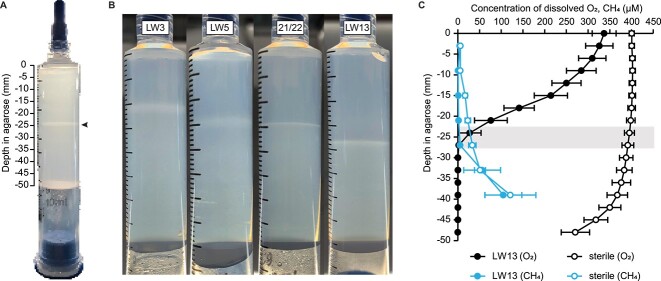
(A) The gradient syringe model ecosystem inoculated with the methanotroph *Methylomonas* sp. strain LW13; arrowhead indicates location of the horizontal band; (B) cultivation of the Lake Washington-isolated methanotrophic strains in the gradient syringe, *Methylosinus* sp. strain LW3 (Day 7 within the syringe), *Methylocystis* sp. strain LW5 (Day 7), *M. tundripaludum* 21/22 (Day 7), and *Methylomonas* sp. strain LW13 (Day 3); images are representative of three independent experiments; (C) Methane and oxygen gradients of inoculated (solid) and sterile (open) syringes after 3 days of incubation show both substrates are depleted at the same depth as the horizontal band (shaded bar) that forms within ~25 mm from the filter tip; data show the mean ± SD of three independent experiments with three technical replicates each.

We established a simple laboratory-based model ecosystem to cultivate methanotrophs using semi-solid agarose in disposable syringes (Fig. 1A). Syringes were equipped with a sterile filter tip and filled with agarose, bacterial inoculum, and NMS medium. We inoculated gradient syringes with individual cultures of four methanotrophs isolated from Lake Washington, Seattle, USA [26]. All methanotrophs inoculated into the gradient syringe formed a horizontal band around the midpoint of the agarose plug in as few as 3 days, even though the agarose was uniformly inoculated with bacteria, a phenomenon previously observed in more complex setups [23, 24]. With additional days of incubation and daily replenishment of methane in the syringe headspace, this band became more opaque.

Different strains of methanotrophs reproducibly formed the band at different rates and depths when they were individually cultured in the gradient syringe (Fig. 1B). The gammaproteobacterium *Methylomonas* sp. LW13 formed a 1-mm-thick band within 3 days of incubation at a depth of ~25 mm from the oxygen side of the syringe. *Methylobacter tundripaludum* 21/22 formed a 0.5-mm-thick band within 4 days of incubation at a depth of 18–24 mm within the gradient syringe. When two methanotrophic alphaproteobacteria were individually cultured in the gradient syringe, they formed bands that were less opaque and more diffuse than the gammaproteobacteria. *Methylosinus* sp. LW3 formed a 2-mm-thick band within 7 days of incubation at a depth of 5–10 mm. *Methylocystis* sp. LW5 formed a band within 4 days at a depth of 14–19 mm and a thickness of 2 mm. Altogether, these findings show the ability to identify reproducible differences between methanotrophic taxa cultured in this system.

### Methane-oxygen counter gradient formation in the syringe model ecosystem

To determine if the syringe model ecosystem contained a methane-oxygen counter gradient, we measured the dissolved oxygen and methane content throughout LW13-inoculated and cell-free syringes after 3 days of incubation. In inoculated syringes, the dissolved oxygen concentration of the agarose closest to the filter tip was 336.9 ± 27.6 μmol/l and steeply declined to <1 μmol/l at the same depth as the horizontal band, around 25 mm from the filter tip (Fig. 1C). Dissolved oxygen remained depleted with a mean concentration of 0.28 ± 0.09 μmol/l for the remainder of the agarose. This dissolved oxygen gradient began to form within 24 h of flushing a methanotroph-inoculated gradient syringe and became steeper over the course of the 3-day incubation period (Fig. S1, see online supplementary material for a color version of this figure).

From the filter tip, the concentration of methane was <2 μmol/l until reaching the depth of the horizontal band, where it then increased in the remaining agarose up to 104.7 ± 42.67 μmol/l in the second-deepest agarose segment (Fig. 1C). In cell-free sterile gradient syringes, a partial oxygen-methane counter gradient formed, but only reached a low of 270.6 ± 32.6 μmol/l dissolved oxygen and 5.79 ± 2.26 μmol/l methane in either gas gradient. These results indicate that bacterial consumption of methane and oxygen drive the formation of the steep methane-oxygen counter gradient in this system and that the intersection of the counter gradient is at the same location as the horizontal band.

### Horizontal band formation is correlated with increased polysaccharide content but not increased cell counts

**Figure 2:**
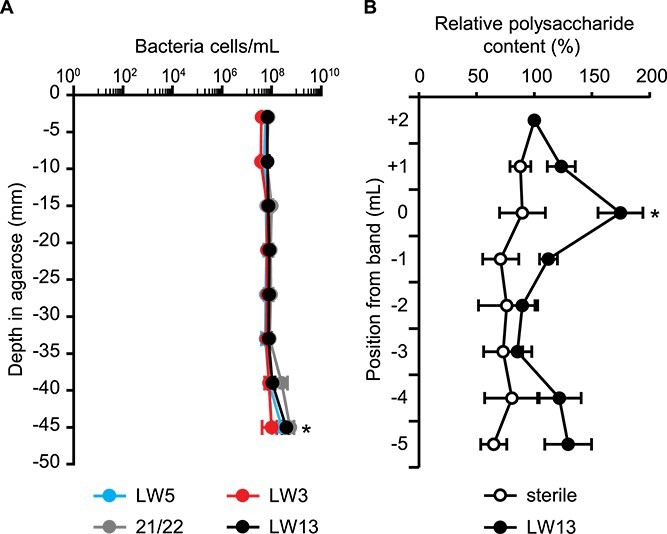
(A) Methanotrophic cell distribution in the gradient syringe one day after band formation; no significant difference in cell counts was observed between segments for any strain, with the exception of the LW13-inoculated syringes containing a significantly higher mean concentration in the deepest segment compared with all other segments (^*^, one-way ANOVA with Tukey–Kramer post hoc analysis, *P* < .05), data show the mean ± SD of two independent experiments with three technical replicates each, and (B) relative polysaccharide levels in LW13-inoculated agarose after 7 days, with absorbance at 490 nm expressed as a percentage relative to the segment nearest the filter tip; the reference point 0 corresponds to the 1-ml segment centered around the horizontal band; significant differences noted from sterile controls at equivalent depths (^*^, two-tailed heteroscedastic *t*-test, α = 0.05); data show the mean ± SD of three independent experiments with two technical replicates each.

We initially hypothesized that the horizontal band contained a larger number of growing cells than the surrounding depths of the syringe. To test this, we quantified the number of cells per ml of agarose for inoculated syringes at different depths for each methanotroph strain using flow cytometry (Fig. 2A). Within 1 day after the appearance of a horizontal band, we extruded 1-ml agarose segments through a needle to determine the concentration of cells per ml. For the methanotrophic strains LW3, LW5, and 21/22, no individual segment contained a significantly higher cells/ml than any other segment from the same syringe. For syringes containing LW13, the deepest segment contained a significantly higher concentration of cells than all other segments within the same syringe. No significant differences were found between the LW13 cell concentrations of the remaining segments, indicating that for all four strains tested, the formation of the horizontal band was not caused by increased cell numbers at that depth. These results were confirmed through counting colony forming units per ml by culturing extruded agarose from syringes inoculated with each strain (Fig. S2, see online supplementary material for a color version of this figure).

Because methanotrophs are known to produce various polysaccharides under different growth conditions [27, 40, 41], we used a colorimetric assay [28, 29] to quantify the relative polysaccharide content at different depths in the gradient syringe. After 7 days of incubating LW13 with daily methane replenishment to increase the overall opacity and thickness of the band, we divided the agarose plug into 1-ml segments, ensuring that the band was centered within a single segment. In agarose segments inoculated with LW13, the segment containing the horizontal band had significantly increased relative polysaccharide content compared with the other segments down to the −3 segment from the band (−33 mm from filter tip) (Fig. 2B). The −5 segment furthest from the filter tip trended upward in relative polysaccharide content, which was consistent with the observation of slightly more biomass at the interface of the agarose plug and the methane-filled headspace. In sterile gradient syringes, only the agarose matrix contributed to the colorimetric response of the assay, and thus, the relative polysaccharide content at increasing depths remained constant. Additionally, we measured the concentration of extracellular DNA and total protein in LW13-inoculated syringes at each depth but did not observe a significant increase in either biomolecule in the band segment (Fig. S3, see online supplementary material for a color version of this figure). Because the polysaccharide content correlated to the observed band position(s) within the gradient syringe, we conclude that the horizontal band formed as a result of increased polysaccharide production by cells positioned at the intersection of the methane-oxygen counter gradient.

### Polysaccharide band formation within the gradient syringe is correlated to changes in gene expression

**Figure 3:**
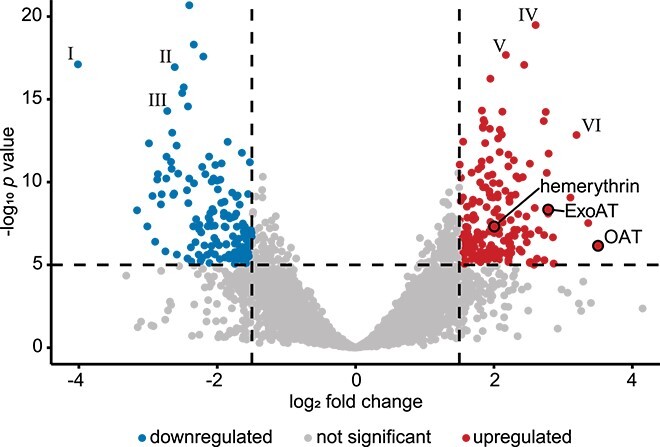
Differentially expressed genes in the band segment compared with the above segment; dashed vertical lines signify cutoffs of |log_2_-fold change| >1.5 and the horizontal line indicates a cutoff of adjusted *P*-value < .0001; highlighted genes predicted to encode: (I) transposase-like protein, (II) heme oxygenase, (III) polyhydroxybutyrate depolymerase, (IV) tryptophan synthase alpha chain, (V) acetyl-CoA carboxylase carboxyl transferase subunit beta, and (VI) ornithine carbamoyltransferase.

To investigate the cellular functions underlying the spatially resolved polysaccharide-producing phenotype observed in the gradient syringe, we compared transcriptomes of LW13 isolated from increasing depths in the agarose matrix. Late exponential/early stationary phase (Fig. S4, see online supplementary material for a color version of this figure) gradient syringes inoculated with LW13 were divided into three 2-ml segments, with the middle segment centered around the polysaccharide band. These segments were designated “above,” “band,” and “below.” Oxygen and methane profiles (Fig. 1C) indicated differing gas substrate ratio for cells in these segments: high oxygen-low methane, low oxygen-low methane, and low oxygen-high methane, respectively. RNA-seq analysis of these segments revealed distinct gene expression profiles, with cells in the band and below segments exhibiting the greatest similarity. Using a threshold of |log_2_(fold change)| >1.5 and adjusted *P* < .0001, we identified 11 differentially expressed genes (DEGs) between the band and the below segments (Fig S5, see online supplementary material for a color version of this figure), and 312 DEGs between the band and the above segments (Fig 3).

In the low oxygen micro-environment of the band segment, methane metabolism genes were relatively downregulated compared with the higher oxygen above segment. The *pmoCAB* methane monooxygenase operon showed an average of 1.26 log_2_-fold decrease in band cells, whereas the *mxaF* and *xoxF* methanol dehydrogenase genes exhibited decreases of 0.623 and 1.02 log_2_-fold change, respectively. However, for all three segments, *pmoCAB* were the most highly expressed among all annotated genes. Annotated ammonium uptake and assimilatory denitrification genes were also upregulated in the band segment. Using less stringent cutoffs, only one DEG was upregulated in the band segment compared with both the above and below segments: a hypothetical protein within a predicted Type VI secretion system gene cluster. In comparing gene expression between band and below segments (Fig. S5A, see online supplementary material for a color version of this figure), there were notably fewer DEGs with a log_2_-fold change >1.5 than in the band vs. above comparison, presumably due to the shared low oxygen micro-environments of the band and below segments. Among 11 upregulated DEGs were two genes predicted to be involved in iron acquisition and storage, and three genes potentially involved in cell division or stress adaptation [42–44].

Comparing the gene expression between cells from the band segment and those from the above segment revealed a large diversity of DEGs with a log_2_-fold change >1.5, highlighting potential differences in cellular activity in these segments (Fig. 3). In the band segment cells, there was significant upregulation of genes annotated as involved in energy conservation, amino acid and nucleotide metabolism/transport, coenzyme metabolism, and carbohydrate and lipid metabolism/transport compared with the above segment, as assigned by the National Center for Biotechnology Information (NCBI) Cluster of Orthologous Genes database [45]. These genes made up 78 of the 177 upregulated genes in the band segment compared with above, suggesting involvement in diverse metabolic activities including energy conservation and synthesis of biomolecules. Additionally, 15 upregulated genes were predicted to be related to cellular structure and motility. Collectively, these upregulated genes suggest intensified activity in metabolism, biosynthesis, translation, transcription, cellular structure, and intracellular processes. Moreover, the increased expression of 17 genes predicted to be involved in intracellular trafficking and secretion, signal transduction, and secondary metabolite biosynthesis that suggests enhanced intracellular communication and responsiveness to external conditions. Using less strict cutoffs, we found several putative functional pathways that were also upregulated, including electron transport, type VI secretion, hopanoid biosynthesis, and cell wall biosynthesis. These results reveal differences in gene expression within this spatially resolved model ecosystem.

### Selected differentially expressed genes are involved in polysaccharide band formation

**Figure 4:**
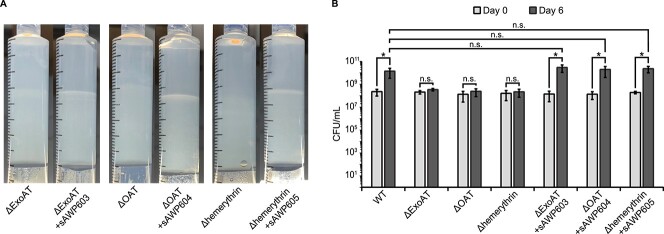
(A) Reduction in polysaccharide band production in three LW13 mutants within the gradient syringe; images are representative of three independent experiments; (B) growth of LW13 strains over 6 days in the gradient syringe; ^*^, significantly different (two-tailed heteroscedastic *t*-test, α = 0.05); n.s., not significantly different, and data show the mean ± SD of three independent experiments with two technical replicates each.

Among the upregulated genes with the greatest log_2_-fold change in the band compared with the above segment, we selected three for further investigation. We chose genes with annotated functions that to our knowledge had not been extensively studied in methanotrophs, especially in the context of polysaccharide production. These genes were predicted to encode a fucose 4-O- acetyltransferase (OAT; gene ID: 2923715777, Integrated Microbial Genomes System [46]), a hemerythrin (2923717515), and an *N*-acyl amino acid synthase of a PEP-CTERM/exosortase system (ExoAT; 2 923 716 464) (Fig. 3). We constructed disruption mutant strains for each of the three genes to determine their individual influence on the growth of LW13 and the formation of the polysaccharide band within the gradient syringe. We disrupted the target genes via electroporation to create the mutant strains ΔOAT, Δhemerythrin, and ΔExoAT. When we individually inoculated these LW13 mutants into gradient syringes, we observed that the mutant strains did not form the same distinct polysaccharide band, and that band formation was restored after gene complementation (Fig. 4A). We also counted the viable cells in each syringe after 6 days of incubation with daily methane replenishment to determine if these genes were involved in cell growth within the system. Although wild-type LW13 grew to more than 75-times its initial CFU/ml concentration in the gradient syringe, the number of viable LW13ΔOAT, LW13Δhemerythrin, and LW13ΔExoAT mutant cells did not significantly increase (*P* < .05) over the same growth period (Fig. 4B). Complementation of the deleted genes led to significantly increased growth in the three mutants from Day 0 to Day 6, restoring final CFU/ml to values comparable to the LW13 wild-type strain. Deletion of these genes did not negatively affect growth in planktonic culture, but instead led to slight but significantly faster growth rates (Fig. S6, see online supplementary material for a color version of this figure). Altogether, these results show that we can use the model gradient syringe system to link genes to phenotypes that would otherwise be missed using conventional culturing methods.

## Discussion

In this work, we demonstrate that culturing methanotrophs within a spatially resolved counter gradient of methane and oxygen can reveal genotype–phenotype associations that are largely unnoticed in conventional culturing approaches. We observed that methanotrophs form a distinct horizontal band located at the intersection of the counter gradient and that it is not due to an increased number of cells at this location, as previously hypothesized [23–25], but instead due to increased polysaccharide content. Using transcriptomics and deletion mutants, we identified three genes (likely out of many) involved in this phenotype. These genes are required for cell growth within the model ecosystem, but their absence did not negatively affect the ability of LW13 to grow in planktonic culture.

The observed increase in polysaccharide content in the horizontal band in the gradient syringe model ecosystem may be due to increased extracellular polymeric substances (EPS) produced by methanotrophs at this depth. An established link exists between methane oxidation, oxygen availability, and EPS production in methylotrophic bacteria [27, 47]. The primary component of this band appears to be polysaccharides, as there were no significant increases in extracellular DNA or total protein (two common EPS components) detected in the band segment (Fig. S3, see online supplementary material for a color version of this figure). However, these results do not negate the possibility that other biomolecules are present, and additional experiments are necessary to determine the exact molecular components of this band. We determined through flow cytometry that cells at the band were comparable in size to those directly adjacent (Fig. S7, see online supplementary material for a color version of this figure). This finding supports the conclusion that the biomolecules contributing to the horizontal band phenotype are likely extracellular and not due to intracellular polysaccharide accumulation or formation of a capsule.

We observed variations in the depth and appearance of the horizontal band when we grew different methanotrophic genera in the gradient syringe (Fig. 1B). This difference may stem from the ability of different methanotrophic genera to adapt to varying oxygen tensions in the environment [14, 17, 18]. It is currently unclear whether the formation of the polysaccharide band is a cause or consequence of this location being at the intersection of the methane-oxygen counter gradient. It is possible that polysaccharide production was higher in cells at the intersection of the counter gradient as a response to low gas concentrations at this location or that polysaccharide production prevented the diffusion of gas substrates once a horizontal band formed. Although the exact triggers for extracellular polysaccharide production in methanotrophs remain to be precisely defined, excess carbon, nitrogen deficiency, and oxygen excess or limitation have all been reported to stimulate its production in methylotrophs [27, 40, 48]. More precisely identifying the involved factors may have practical implications for applied methanotrophy, such as methane mitigation in landfill covers and biofilters, which are negatively affected by the production of EPS [47].

Transcriptomic analysis of different segments within an LW13-inoculated gradient syringe revealed broad global gene expression differences, with the band and below segments being most similar (Fig. S5A, see online supplementary material for a color version of this figure), suggesting that oxygen availability is a major influence on cellular heterogeneity. Methane monooxygenase gene expression varied between the examined syringe segments but remained highly expressed in LW13, illustrating methanotrophs’ adaptability to oxygen changes. Because cells throughout the gradient syringe appeared to have comparable growth rates and sufficient metabolic activity, we aimed to link genes upregulated in the band segment with the polysaccharide phenotype. Previously, the role of the exosortase protein EpsH in production of EPS in LW13 was identified [49] and confirmed [50], where disruption of *epsH* resulted in significantly reduced EPS in ammonium mineral salts media. However, this gene was not significantly differentially expressed by cells of any segment in our RNA-seq analysis, possibly due to nitrate, rather than ammonium, being used as the nitrogen source in our experiments.

To explore additional potential gene-phenotype connections, we investigated a subset of genes that were upregulated in the band segment that had not yet been experimentally linked to polysaccharide production in methanotrophs. *N*-acyl amino acid synthases have been phylogenetically linked to the PEP-CTERM/exosortase system (ExoAT) [51], a putative protein sorting system in Gram-negative bacteria associated with EPS production [52]. The predicted product of the fucose 4-O-acetylase like acetyltransferase gene (OAT) likely engages in acylation processes impacting various sugar-containing cell-surface structures, including exopolysaccharides [53]. Hemerythrin is a non-heme oxygen-binding protein that serves as an oxygen scavenging transporter for particulate methane monooxygenase-containing aerobic methanotrophs such as LW13 and was predictably upregulated in the relatively lower oxygen concentrations of the band segment to the above segment in our study [15].

Although ExoAT and OAT are not considered essential to cell survival and have clear potential links to the production of extracellular polysaccharides, individual knockouts of these genes in LW13 had a negative effect on both horizontal band formation and growth (Fig. 4) in the gradient syringe but did not interfere with growth in planktonic culture (Fig. S6, see online supplementary material for a color version of this figure). The fact that band formation and growth in the gradient syringe were both affected in each of these mutants suggests that either methanotrophic growth is dependent on horizontal band formation, or that the formation of the band is dependent on sufficient cell growth. Both reduction in band formation and growth in the gradient syringe by the hemerythrin mutant suggests that sufficient cell growth or metabolic activity may be required for band production, as hemerythrin production is closely linked with methane oxidation activity in micro-oxic environments [54]. Discovery of a gene that when deleted only affects band formation but not growth in the gradient syringe would further clarify the nature of this behavior.

Our results have raised questions about methanotroph physiology within a methane-oxygen counter gradient that can now be studied at the molecular level using this model ecosystem. Extending this concept, our laboratory-based model ecosystem can be used to simultaneously cultivate multiple strains isolated from a methane-oxidizing bacteria community to study the molecular and genetic-level details of chemical and physical interactions between strains. Alternatively, the simple design of the gradient syringe could be modified to cultivate other types of bacteria that exist at the boundary between oxygen and non-oxygen gas environments. This includes examples such as the aerobic H_2_-oxidizing “Knallgas” bacteria present in termite guts [6, 55], and the soil bacteria from the genus *Bradyrhizobium* that aerobically oxidize carbon monoxide generated by legume roots [56, 57]. By studying these biogeochemically relevant microbes in a model ecosystem that mimics their respective gas counter gradients, we can gain deeper insights into their unique physiology and metabolic adaptations. Overall, our study not only sheds light on the behavior of methanotrophs but also underscores the broader applicability of simple laboratory-based model ecosystems for linking genes with organismal phenotypes.

## Supplementary Material

GradientSyringe_SupplementaryInfo_rev20240219-1_wrae060

## Data Availability

The datasets generated and analyzed during the current study are available in the NIH NCBI Gene Expression Omnibus database under accession number GSE243827. This includes raw transcriptomic data, as well as normalized counts and computed pairwise fold changes for the different syringe segments.
