# Supplementary material for: Linking methanotroph phenotypes to genotypes using a simple spatially resolved model ecosystem
Source: ISME J. 2024 Apr 16;18(1):wrae060. doi: 10.1093/ismejo/wrae060 (PMC11072679; doi:10.1093/ismejo/wrae060)
Supplement: GradientSyringe_SupplementaryInfo_rev20240219-1_wrae060 [file gradientsyringe_supplementaryinfo_rev20240219-1_wrae060.pdf]

**SUPPLEMENTARY INFORMATION**

# Linking methanotroph phenotypes to genotypes using a simple spatially resolved model ecosystem

Delaney G. Beals and Aaron W. Puri[#]

Department of Chemistry and the Henry Eyring Center for Cell and Genome Science, University of Utah, Salt Lake City, Utah, USA

Keywords: methanotroph, methane, model ecosystem, phenotypic heterogeneity

#Corresponding author:
Aaron W. Puri
315 S 1400 E Rm 2020
Salt Lake City, UT 84112
USA
(801) 213-1408
a.puri@utah.edu

**SUPPLEMENTARY FIGURES**

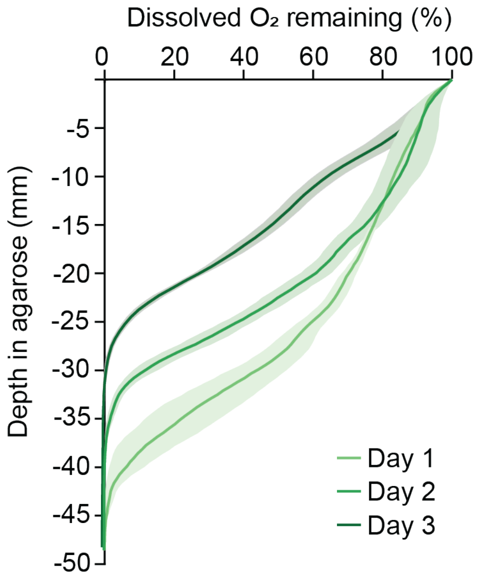


**Figure S1.** Development of the oxygen gradient in LW13-inoculated gradient syringes replenished daily with methane over three days. Data show the mean ± standard deviation (shaded areas) of three independent experiments with five technical replicates each.

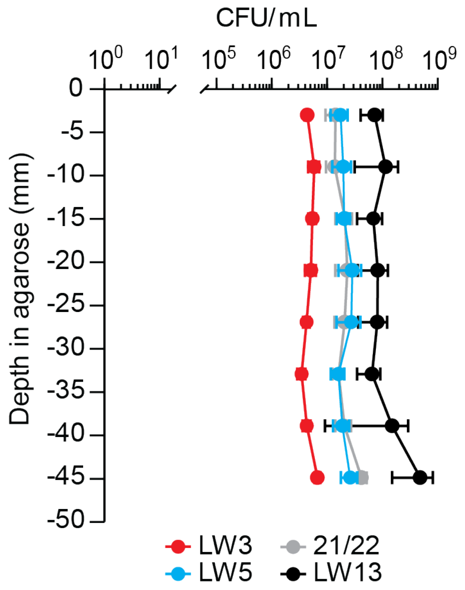


**Figure S2.** Distribution of viable methanotrophic cells in the gradient syringe one day after band formation by viable cell counts (CFU/mL). Data show the mean ± standard deviation of three independent experiments with five technical replicates each. The mean CFU/mL from any segment of any strain is not significantly different than any other segment mean CFU/mL of the same strain; one-way ANOVA with $\alpha = 0.05$.

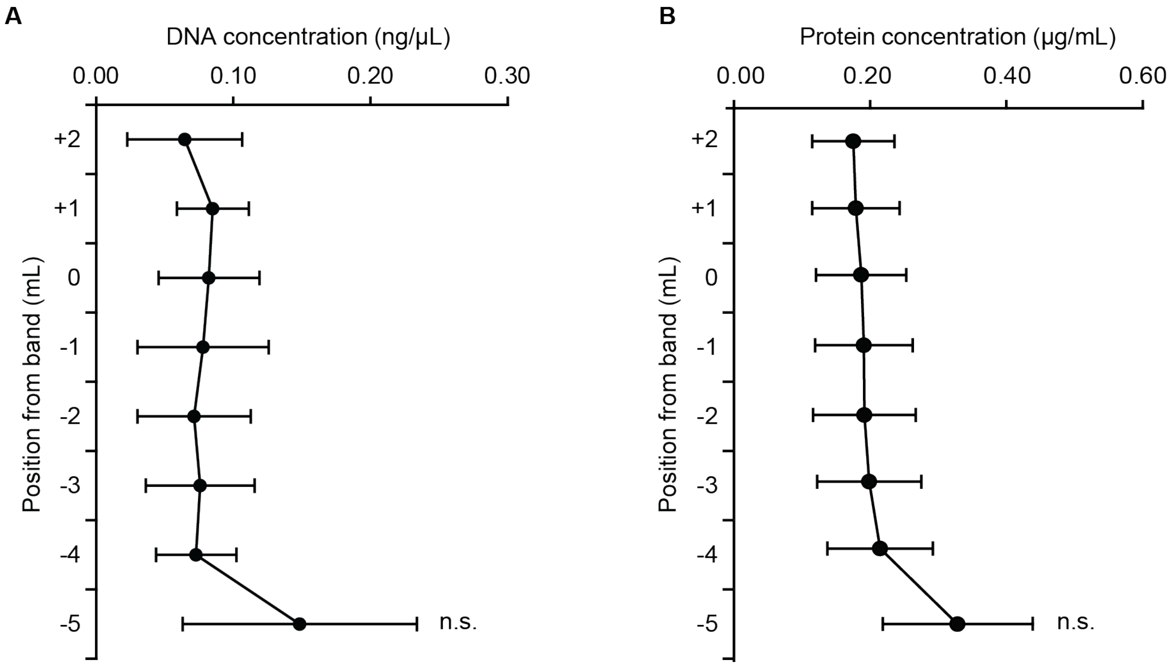


**Figure S3.** Distribution of **(A)** Extracellular DNA concentration and **(B)** protein concentration in LW13-inoculated syringes incubated for seven days. Data points and error bars represent the mean ± standard deviation of two technical replicates from two independent experiments; n.s., not significant; one-way ANOVA with $\alpha = 0.05$.

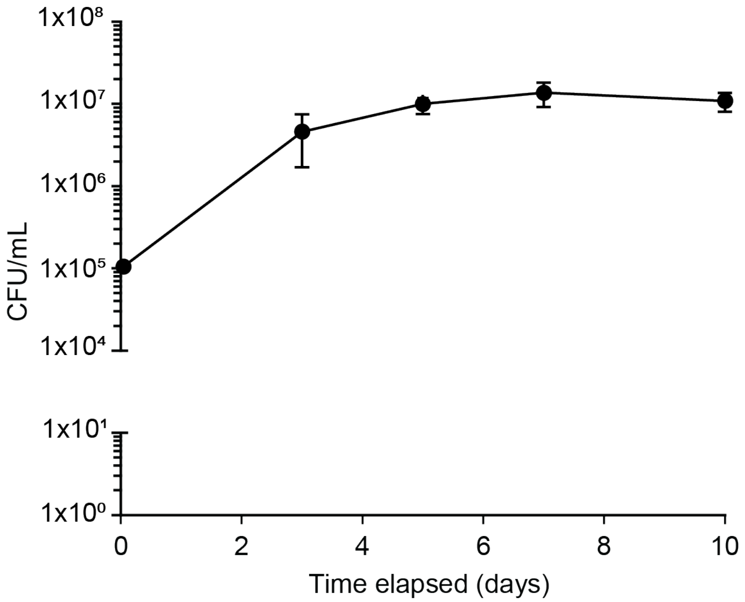


**Figure S4.** Viable cell counts of entire agarose plug of LW13-inoculated gradient syringes over ten days. Data show the mean ± standard deviation of three independent experiments with two technical replicates each.

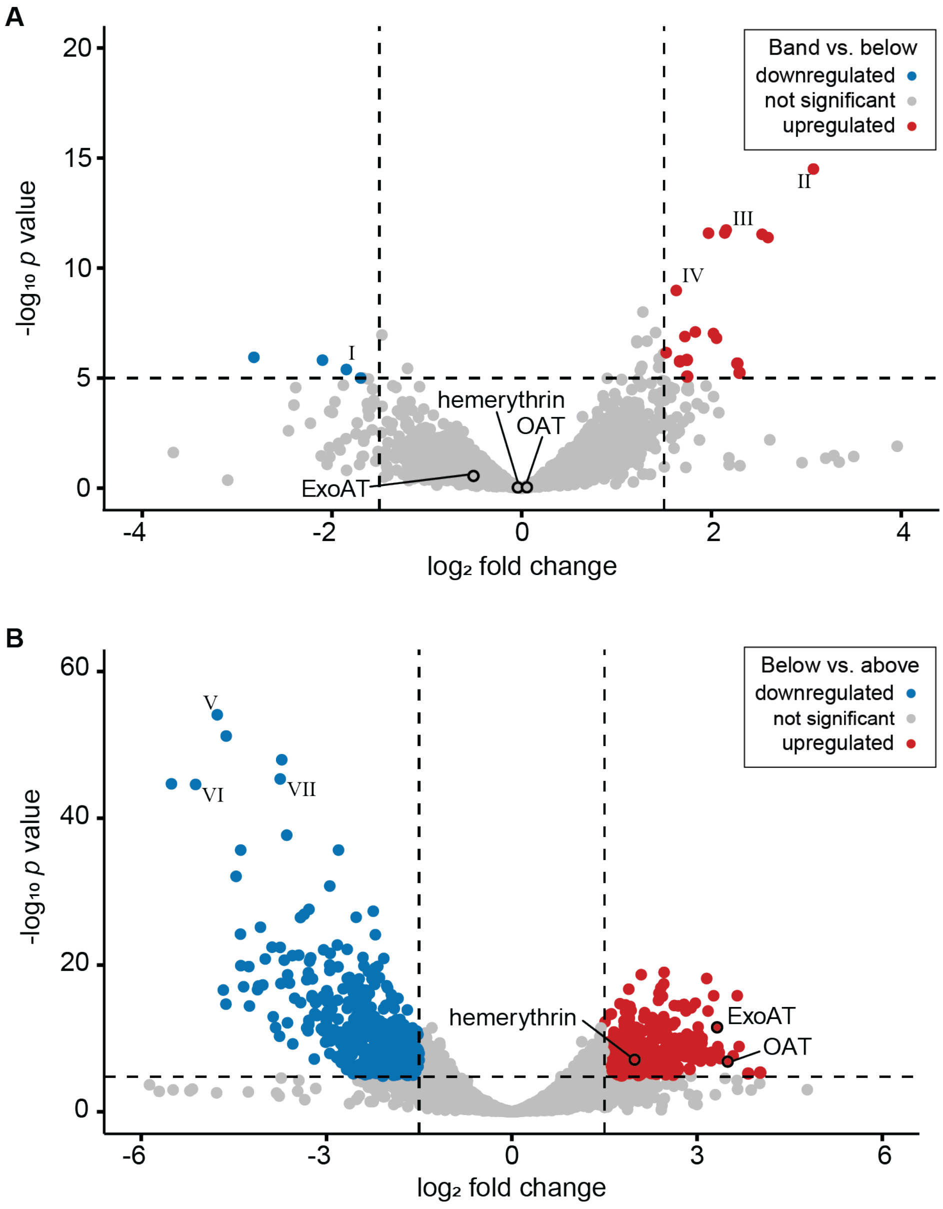


**Figure S5.** Differentially expressed genes comparing different gradient syringe segments. Dashed vertical lines signify cutoffs of |$\log_2$-fold change| >1.5 and the horizontal line indicates a cutoff of adjusted *p* value <0.0001. **(A)** Band vs. below comparison with highlighted significantly differentially expressed genes predicted to encode (I)FMN-dependent NADH-azoreductase, (II) bacterioferritin-associated ferredoxin, (III) heme oxygenase, (IV) cell division protein FtsA. **(B)** Below vs. above comparison with highlighted significantly differentially expressed genes predicted to encode (V) heme oxygenase, (VI) bacterioferritin-associated ferredoxin, (VII) two-component response regulator RegA .

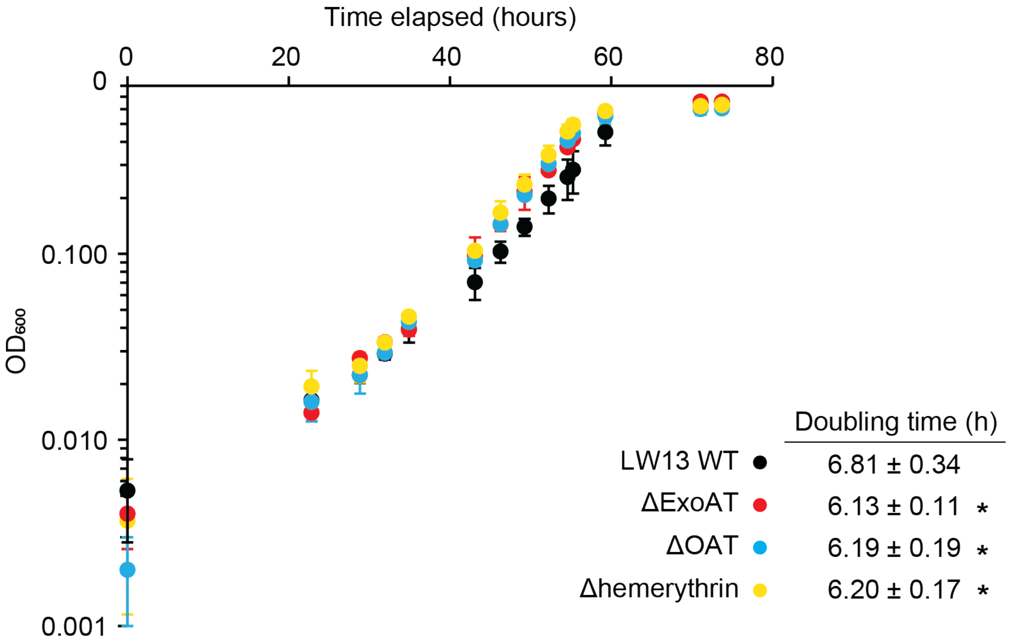


**Figure S6.** Growth curves of wild-type LW13 and single deletion mutants in shaken liquid nitrate mineral salts (NMS) media at room temperature. Doubling times in hours are shown for strains in liquid culture; mutant doubling times were significantly lower than the wild-type control (one-way ANOVA with Tukey-Kramer post hoc analysis, $p < 0.05$). Data points and values represent the mean ± standard deviation of three technical replicates and are representative of three independent experiments.

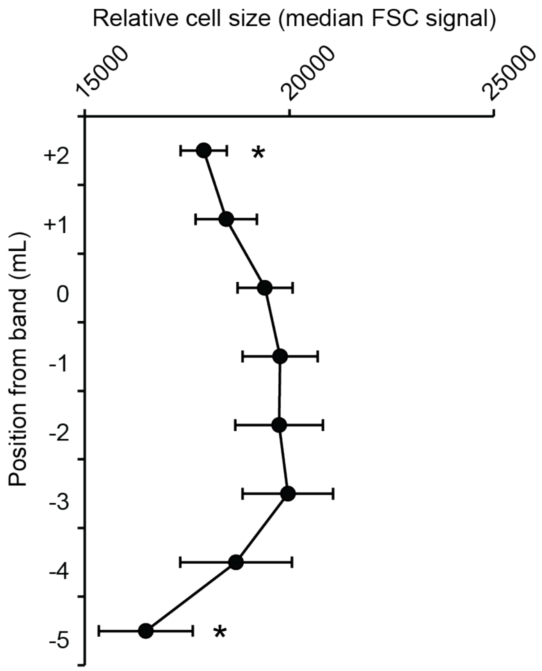


**Figure S7.** Relative cell size of LW13 in gradient syringe segments was assessed after seven days of incubation using flow cytometry, focusing on the mean ± standard deviation of the median forward scatter (FSC) signal from each segment. *, significantly different ($p$ <0.01) from the overall mean; one-sample $t$-test with Bonferroni correction for multiple comparisons. Data are from three independent experiments, each with four technical replicates.

## SUPPLEMENTARY TABLES

**Table S1.** Strains used in this study.

| **Strain** | **Puri Lab strain collection name** | **Description** | **Source or reference** |
|---|---|---|---|
| *Methylomonas* sp. strain LW13 | AWP145 | Aerobic methane-oxidizing bacterium isolated from Lake Washington sediment | DOI: 10.1128/genomeA.00103-15 |
| *Methylobacter tundripaludum* strain 21/22 | AWP100 | Aerobic methane-oxidizing bacterium isolated from Lake Washington sediment | DOI: 10.1128/genomeA.00103-15 |
| *Methylosinus* sp. LW3 | AWP131 | Aerobic methane-oxidizing bacterium isolated from Lake Washington sediment | DOI: 10.46936/10.25585/60007343 |
| *Methylocystis* sp. LW5 | AWP132 | Aerobic methane-oxidizing bacterium isolated from Lake Washington sediment | DOI: 10.46936/10.25585/60007343 |
| *Methylomonas* sp. strain LW13ΔOAT | AWP491 | Strain containing deletion of gene 2923715777 with predicted fucose 4-O-acetylase-like acetyltransferase product | Locus tag: Ga0485366_01_330073_331065 (https://img.jgi.doe.gov) |
| *Methylomonas* sp. strain LW13Δhemerythrin | AWP492 | Strain containing deletion of gene 923717515 with predicted hemerythrin product | Locus tag: Ga0485366_01_2188896_2189291 |
| *Methylomonas* sp. strain LW13ΔExoAT | AWP493 | Strain containing deletion of gene 2923716464 with predicted N-acyl amino acid synthase of PEP-TERM/exosortase system product | Locus tag: Ga0485366_01_1079880_1080629 |
| *Methylomonas* sp. strain LW13ΔOAT::OAT | AWP494 | ΔOAT with OAT under *nptII* promoter inserted between 2923716887 and 2923716888 | This study |
| *Methylomonas* sp. strain LW13Δhemerythrin::hemerythrin | AWP495 | Δhemerythrin with hemerythrin under *nptII* promoter inserted between 2923716887 and 2923716888 | This study |
| *Methylomonas* sp. strain LW13ΔExoAT::ExoAT | AWP496 | ΔExoAT with ExoAT under *nptII* promoter inserted between 2923716887 and 2923716888 | This study |

**Table S2.** SOE constructs used in this study.

| Construct | Description |
|---|---|
| sAWP507 | SOE construct to create LW13ΔOAT; kanR cassette flanked by ~800 bp regions directly adjacent to 2923715777 |
| sAWP508 | SOE construct to create LW13Δhemerythrin; kanR cassette flanked by ~800 bp regions directly adjacent to 2923717515 |
| sAWP510 | SOE construct to create LW13ΔExoAT; kanR cassette flanked by ~800 bp regions directly adjacent to 2923716464 |
| sAWP603 | SOE construct to complement LW13ΔExoAT with 2923716464 under *nptII* promoter between 2923716887 and 2923716888 in genome |
| sAWP604 | SOE construct to complement LW13ΔOAT with 2923715777 under *nptII* promoter between 2923716887 and 2923716888 in genome |
| sAWP605 | SOE construct to complement LW13Δhemerythin with 2923717515 under *nptII* promoter between 2923716887 and 2923716888 in genome |

**Table S3.** Cloning primers used in this study.

| Primer name | Sequence (5' to 3')* | Description |
|---|---|---|
| **Gene deletion constructs** | | |
| oAWP1238 | GATGAGAGCTTTGTTGTAGG | For amplification of kanR cassette for insertion between flanks to replace native gene |
| oAWP1239 | TCTCGAGTCCCGTCAAGTC | |
| oAWP1646_507U_fwd | AGACGACATCGAATTTAAACTTCGC | For amplification of flanks to construct sAWP507 to create LW13ΔOAT |
| oAWP1647_507U_rev | AACTGGTCCACCTACAACAAAGCTCTCATCGCCCCAATACAACAAGGAAAATTGCA | |
| oAWP1648_507D_fwd | AGCATTACGCTGACTTGACGGGACTCGAGATGTGCCATTATTGAACTCTGTGACA | |
| oAWP1649_507D_rev | GCACACCTATCACGCCATTGCT | |
| oAWP1650_508U_fwd | GCAAAACCTGGGCGACGATTGC | For amplification of flanks to construct sAWP508 to create LW13Δhemerythrin |
| oAWP1651_508U_rev | AACTGGTCCACCTACAACAAAGCTCTCATCGGTTTGGTGCTCTTGGTCGGCA | |
| oAWP1652_508D_fwd | AGCATTACGCTGACTTGACGGGACTCGAGATCGACATGGCGTATTCTGAAGCA | |
| oAWP1653_508D_rev | AATTGGGCACGTTACGCGGGTC | |
| oAWP1658_510U_fwd | GCGGCGATGCCTCTGTGTTTCA | For amplification of flanks to construct sAWP510 to create LW13ΔExoAT |
| oAWP1659_510U_rev | AACTGGTCCACCTACAACAAAGCTCTCATCTGCGTTTGCTTTCAGGCGTGTCA | |
| oAWP1660_510D_fwd | AGCATTACGCTGACTTGACGGGACTCGAGAGAGCAATCGGTGGCTAAGCGGC | |
| oAWP1661_510D_rev | ACCACACGGCAAGCGCTAAAGC | |
| **Gene complementation constructs** | | |
| oAWP1877_2887_fwd | ACCATTAACGGCGAAGTCAGCA | for amplification of 2923716888 from LW13 genome; overlaps with *nptII* promoter |
| oAWP1927_2887_pnpt_rev | TCCCCAATTCCTGGCAGTTTATGGGTCAATCCGAGTACCACTACAGAGCT | |
| oAWP1928_pnpt_2887_fwd | ATTAGTTGTAAGCTCTGTAGTGGTACTCGGATTGACCCATAAACTGCCAG | for amplification of *nptII* promoter; 30 nt overlap with 2923716888 |
| oAWP1929_pnpt_exo_rev | ACTATCAAATGAATGCTTTTCAGAAATCAATTTTTCTTCCTCCACTAGTA | for amplification of *nptII* promoter; 30 nt overlap with ExoAT |
| oAWP1931_pnpt_fuc_rev | TATATCAACGAGTCTGTTTTTTTTATCCAATTTTTCTTCCTCCACTAGTA | for amplification of *nptII* promoter; 30 nt overlap with OAT |
| oAWP1933_pnpt_heme_rev | TTGAGCCGCAGTCCAAGTAATTAAAGCCATTTTTTCTTCCTCCACTAGTA | for amplification of *nptII* promoter; 30 nt overlap with hemerythrin |
| oAWP1930_exo_pnpt_fwd | AGAGACAGGATACTAGTGGAGGAAGAAAAATTGATTTCTGAAAAGCATTC | for amplification of ExoAT; 30 nt overlap with *nptII* promoter |
| oAWP1888_589_zeo_rev | TGGCCATAGCTGTTTCCTGTGTGAATACCTTTAAGCTCTTTGCCGCTTAG | for amplification of ExoAT; 30 nt overlap with zeoR insert |
| oAWP1932_fuc_pnpt_fwd | AGAGACAGGATACTAGTGGAGGAAGAAAAATTGGATAAAAAAAACAGACT | for amplification of OAT; 30 nt overlap with nptII promoter |
| oAWP1890_590_zeo_rev | TGGCCATAGCTGTTTCCTGTGTGAATACCTCTAATTTGTCACAGAGTTCAATAATG | for amplification of OAT; 30 nt overlap with zeoR insert |

| | | |
|---|---|---|
| oAWP1934_heme_pnpt_fwd | AGAGACAGGATACTAGTGGAGGAAGAAAAAATGGCTTTAATTACTTGGAC | for amplification of hemerythrin; 30 nt overlap with *nptII* promoter |
| oAWP1892_591_zeo_rev | TGGCCATAGCTGTTTCCTGTGTGAATACCTTTACTTCAATGCTTCAGAATACGC | for amplification of hemerythrin; 30 nt overlap with zeoR insert |
| oAWP1883_zeo_589_fwd | CAATCGGTGGCTAAGCGGCAAAGAGCTTAAAGGTATTCACACAGGAAACA | for amplification of zeoR; 30 nt overlap with ExoAT |
| oAWP1884_zeo_590_fwd | GTGCCATTATTGAACTCTGTGACAAATTAGAGGTATTCACACAGGAAACA | for amplification of zeoR; 30 nt overlap with OAT |
| oAWP1885_zeo_591_fwd | GACATGGCGTATTCTGAAGCATTGAAGTAAAGGTATTCACACAGGAAACA | for amplification of zeoR; 30 nt overlap with hemerythrin |
| oAWP1886_zeo_2888_rev | CAGGCAAAAAAAAGCCCGCTGTATCGCGTATTATTCAGTCCTGCTCCTCG | for amplification of zeoR; 30 nt overlap with 2923716887 |
| oAWP1881_2888_zeo_fwd | ACTTCGTGGCCGAGGAGCAGGACTGAATAATACGCGATACAGCGGGCTTT | for amplification of 2923716887 from LW13 genome; overlaps with zeoR |
| oAWP1882_2888_rev | AAATTTTTACCCAAACTGCTTGGTCTC | |

*Homology regions used for SOE PCR amplification are underlined.

## SUPPLEMENTARY REFERENCES


1. Kalyuzhnaya MG, Lamb AE, McTaggart TL, Oshkin IY, Shapiro N, Woyke T, Chistoserdova L. Draft Genome Sequences of Gammaproteobacterial Methanotrophs Isolated from Lake Washington Sediment. *Genome Announc* 2015; **3**: e00103-15.
